# Larvicidal and repellent potential of *Ageratum houstonianum* against *Culex pipiens*

**DOI:** 10.1038/s41598-022-25939-z

**Published:** 2022-12-10

**Authors:** Doaa El Hadidy, Abeer M. El Sayed, Mona El Tantawy, Taha El Alfy, Shaimaa M. Farag, Doaa R. Abdel Haleem

**Affiliations:** 1 Department of Medicinal Plants and Natural Products, National Organization for Drug Control and Research (NODCAR), 51-Wezaret El-Zeraa St, Giza, 12611 Egypt; 2 Department of Pharmacognosy, Faculty of Pharmacy, Cairo University, Kasr El Aini, 11562 Egypt; 3 Department of Entomology, Faculty of Science, Ain Shams University, Cairo, 11566 Egypt

**Keywords:** Developmental biology, Drug discovery, Ecology

## Abstract

Mosquitoes are unquestionably the most medic arthropod vectors of disease. *Culex pipiens*, usually defined as a common house mosquito, is a well-known carrier of several virus diseases. Crude ethanol extracts of different organs of *Agratum houstonianum* are tested with *Culex pipiens* Linnaeus (Diptera: Culicidae) to determine their larvicidal, antifeedant, and repellency effects. Alongside biochemical analysis, the activity of the AChE, ATPase, CarE, and CYP-450 is detected in the total hemolymph of the *C. pipiens* larvae to examine the enzymatic action on the way to explain their neurotoxic effect and mode of action. Through HPLC and GC–MS analysis of the phytochemical profile of *A. houstonianum* aerial parts is identified. The larvicidal activity of aerial parts; flower (AF), leaf (AL), and stem (AS) of *A. houstonianum* extracts are evaluated against the 3rd instar larvae of *C. pipiens* at 24-, 48- and 72-post-treatment. *A. houstonianium* AF, AL, and AS extracts influenced the mortality of larvae with LC50 values 259.79, 266.85, and 306.86 ppm, respectively after 24 h of application. The potency of AF and AL extracts was 1.69- and 1.25-folds than that of AS extract, respectively. A high repellency percentage was obtained by AF extract 89.10% at a dose of 3.60 mg/cm^2^. *A. houstonianium* AF prevailed inhibition on acetylcholinesterase and decrease in carboxylesterase activity. Moreover, a significant increase in the ATPase levels and a decrease in cytochrome P-450 monooxegenase activity (− 36.60%) are detected. HPLC analysis prevailed chlorogenic and rosmarinic acid as the major phenolic acids in AL and AF, respectively. GC–MS analysis of *A. houstonianum* results in the identification of phytol as the major makeup. Precocene I and II were detected in AF. Linoleic, linolenic, and oleic acid were detected in comparable amounts in the studied organs. Overall, results suggest that the *A. houstonianum* flower extract (AF) exhibits significant repellent, antifeedant, and larvicidal activities.

## Introduction

Mosquitoes considered vectors to a wide variety of serious human diseases. The *Culex pipiens* is widely distributed in Egypt causing nuisance to humans and transmits several viral diseases^1^. It is the vector of West Nile virus^2^, Rift Valley fever virus^3^, *Wuchereria bancrofti*^4^, yellow fever^5^, filariasis^6^ and other major public health problems worldwide which cause a significant human and animal mortality and morbidity in addition to sever economic losses. The mosquito control mainly based on the application of synthetic insecticides as larvicides or as adult repellents^7^. The chemical insecticides have adverse impacts on the health and environment beside to the development of resistance^8^. There is global interest in developing natural products as alternatives to conventional insecticides for mosquito control^9^. Many plant species have been screened for their repellent and insecticidal property^10^. Family Asteraceae contained many plant species which have been described for their medicinal and insecticidal purposes^11^. *Ageratum houstonianum* Mill. belonging to this family is a medicinal plant and possesses antimicrobial activity^10^. There are some previous reports on the insecticidal activities^12^ of the different extracts of leaves of *A. houstonianum* as well as repellency against mosquitoes^13^. Furthermore, *A. houstonianum* has found to be a potent source of natural antioxidants^14^. Several classes of compounds were reported from *A. houstonianum*^15–19^. However, a literature survey has shown that there is no report on the phytochemicals of ethanolic extracts of different aerial parts (leaves, stems and flowers) of the Egyptian *A. houstonianum* which prompted authors to investigate the secondary metabolite profiles of the different organs under study. This study was planned to evaluate the larvicidal activity, repellant and antifeedant efficiency of ethanolic extracts of different aerial parts of *A. houstonianum* against *C. pipiens* larvae and adult. As well as study their enzymatic action to explain their neurotoxic effect and mode of action. Alongside investigation of the lipoidal and polyphenolic phytochemical profile through GC–MS and HPLC analysis were carried out respectively, to shed light on the bioactive components of different organs of *A. houstonianum* to which the biological activities may be attributed.

## Results

### Determination of the total phenolic contents

Quantitative determination of phenolic contents of *A. houstonianum* AL, AS, and AF extracts were determined. It was observed that the ethanolic extract of the AF have the highest total phenolic content, followed by the AL then the AS with values of 5.65, 4.82 and 3.39 µg GAE/mg respectively. Flower was the richest extract in the flavonoid contents with value 5.07 µg QE/mg. Whereas the leaves have half the flavonoid content of the flower.

### HPLC analysis and identification of phenolic compounds

HPLC analysis of 70% alcoholic extract of AL, AS, and AF were expressed as (mg/g) extract and complied in Table 1 and the chromatograms are presented in Fig. S1. Allowed identification and quantification of several phenolic acid and flavonoids. It was observed that the total identified phenolic acids in extract of AL, AS, and AF were 8.35, 2.64 and 12.89 mg/g extract, respectively. Chlorogenic acid is the major one among the total phenolic acids by7.12, 5.19, 1.99 mg/g in AL, AF and AS, respectively. Rosmarinic acid was also detected at high concentration in the AF 7.303 mg/g while it was detected in small amount in the AL and AS 0.77 and 0.49 mg/g, respectively. On the other hand, 14 flavonoids were identified 1.32, 0.48 and 6.43 mg/g extract for the AL, AS and AF, respectively. Rutin was detected at high concentration as 0.92, 0.52, and 0.33 mg/g in AL, AF and AS respectively. Also, apigenin was found in the flowers extract at a concentration of 1.79 mg/g.

**Table 1:** HPLC analysis of the alcoholic extracts of leaves, stems, and flowers of *A. houstonianum*.

Peak no.	Retention time (min)	Identified compound	Conc. (mg/g extract)		
			L	S	F
1	3.9	Gallic	0.08	0.01	0.01
2	7.7	Protocatechuic	0.09	0.09	0.03
3	12.1	*p*-hydroxybenzoic	0.08	0.00	0.08
4	12	Gentisic	–	–	–
5	15.2	Catechin	–	–	–
6	16.5	Chlorogenic	7.12	1.99	5.20
7	17.2	Caffeic	0.07	0.02	0.13
8	19.3	Syringic	0.01	–	0.04
9	21.2	Vanillic	0.01	–	0.04
10	28.9	Ferulic	0.01	–	0.00
11	30.7	Sinapic	0.02	–	0.02
12	35	*p*-coumaric	0.08	0.01	0.05
13	34.5	Rutin	0.93	0.34	0.53
14	38.2	Apigenin-7-glucoside	0.13	0.08	3.85
15	39.2	Rosmarinic	0.78	0.49	7.30
16	46.9	Cinnamic	0.01	0.02	0.01
17	49.5	Qurecetin	0.02	–	0.03
18	55.2	Apigenin	0.17	0.06	1.80
19	55.9	Kaempferol	0.02	0.02	0.15
20	59	Chrysin	0.04	0.00	0.08
Total identified phenolic acids			8.35	2.64	12.89
Total identified flavonoids			1.32	0.49	6.43

*L* leaves, *S* stems, *F* flowers, – not identified.

### GC/MS analysis of the lipoidal contents

It was concluded that the yield of lipoidal matter of leaves, stems, and flowers were (3.3%, 1.2% and 4.7%), respectively. The percentage of the unsaponifiable matter (USM) were (58.80%, 55.20% and 58.10%) and FAME were (38.20%, 33.70% and 40.40%) in the extracts of leaves, stems, and flowers, respectively. GC/MS analysis leads to identification of 30, 26 and 31components representing (99.27%, 99.33% and 97.50%) of the *n*-hexane extract yield of leaves, stems and flowers respectively (Table 2, Fig. S2). It was observed that: unsaponifiable matter was composed of hydrocarbons, alcohols, ketones, aldehydes, esters, acids, phenols, sterols, chromenes, quinones, lactones and epoxides. The hydrocarbons represented (19.06%, 10.77 and 15.24%) of the USM of leaves, stems, and flowers respectively. The main of which was 5-Octadecene (3.07%) in leaves, 3-Eicosene (2.78%) in stem and in flowers was Tridecane, 5-methyl (6.92%). Alcohols were the major identified class of compounds of USM of the leaves, stems and flowers representing (41.48%, 60.96% and 26.60%, respectively).

**Table 2:** GC/MS analysis of the unsaponifiable matter (USM) of *n*-hexane extract of the leaves, stem and flowers of *A. houstonianum*.

Peak no.	Rt (min)	Identified compound	Molecular formula	Base peak	Molecular ion peak (M^+^)	Yield (%)		
						L	S	F
1	13.91	3-Butylcyclohexanone	C_10_H_18_O	55	154	0.79	–	–
2	19.20	5-Tetradecene	C_14_H_28_	55	196	–	0.66	–
3	19.21	3-Tridecene	C_13_H_26_	41	182	1.54	–	–
4	21.16	2,6-Dibutyl-2,5-cyclohexadiene-1,4-dione	C_14_H_20_O_2_	41	220	–	–	0.40
5	21.37	2,6-Di(t-butyl)-4-hydroxy-4-methyl-2,5-cyclohexadien-1-one	C_15_H_24_O_2_	57	236	–	–	0.65
6	22.42	2-Allyl-5-t-butylhydroquinone	C_13_H_18_O_2_	191	206	–	1.49	–
7	22.43	Phenol,2,4*bis* (1,1dimethyl ethyl)	C_14_H_22_O	191	206	1.94	–	–
8	22.46	7-Methoxy-2,2,8-trimethyl chromene	C_13_H_16_O_2_	189	204	–	–	0.65
9	23.96	3-Hexadecene	C_16_H_32_	55	224	–	1.73	–
10	23.97	7-Hexadecene	C_16_H_32_	55	224	2.98	–	–
11	25.23	Caryophyllene oxide	C_15_H_24_O	41	220	–	–	3.21
12	25.80	2H-1-Benzopyran,6,7-dimethoxy-2,2-dimethyl **(precocene II)**	C_13_H_16_O_3_	205	220	22.08	13.26	19.20
13	26.20	7-t-Butyl-3,3-dimethyl-1-indanone	C_15_H_20_O	201	216	–	–	6.05
14	26.29	α-Bisabolol	C_15_H_26_O	43	222	–	–	0.54
15	26.32	Heptadecane	C_17_H_36_	57	240	1.90	1.24	–
16	26.38	Ledene oxide	C_15_H_24_O	43	220	–	–	0.52
17	27.12	Methyl 1,5-di-tert-butylbenzene-3-carboxylate	C_16_H_24_O_2_	233	248	–	–	1.28
18	28.10	Loliolide	C_11_H_16_O_3_	43	196	–	1.12	0.49
19	28.28	5-Octadecene	C_18_H_36_	55	252	3.07	1.91	–
20	28.41	Octadecane	C_18_H_38_	43	254	0.61	–	–
21	28.84	Erythro-(*cis*)(1,4),(cis)(1′,4′)-4,4′-Dihydroxybicyclooctyl	C_16_H_30_O_2_	67	254	–	0.67	–
22	28.85	Cyclooctenone, dimer	C_16_H_24_O_2_	55	248	0.72	–	–
23	28.90	α-Bisabolene epoxide	C_15_H_24_O	43	220	–	–	0.44
24	28.97	11,13-Dihydro-11áH-arbusculin B	C_15_H_22_O_2_	219	234	1.09	1.43	–
25	29.23	3,7,11,15-Tetramethyl-2-hexadecen-1-ol	C_20_H_40_O	81	296	2.21	1.41	4.93
26	29.38	2-Pentadecanone,6,10,14-trimethyl	C_18_H_36_O	43	268	2.05	4.69	2.13
27	30.15	2-Methoxymethyl-4,4-dimethyl-5-phenyldihydropyran	C_15_H_20_O_2_	43	232	–	–	0.83
28	30.73	2-Methylhexadecanal	C_17_H_34_O	58	254	–	0.82	–
29	31.02	Hexadecanoic acid, methyl ester	C_17_H_34_O_2_	74	270	–	–	0.38
30	32.18	5-Eicosene	C_20_H_40_	55	280	2.51	2.78	0.48
31	32.27	Eicosane	C_20_H_42_	57	282	–	0.82	–
32	32.28	Pentadecanoic acid, 2,6,10,14-tetramethyl, methyl ester	C_20_H_40_O_2_	43	312	0.95	–	–
33	32.99	Nerolidol	C_15_H_26_O	41	222	0.99	–	–
34	33.02	Geranyl linalool	C_20_H_34_O	69	290	–	–	1.35
35	33.22	Cembrene	C_20_H_32_	68	272	–	0.77	
36	33.83	Acetic acid, 3,7,11,15-tetramethyl-hexadecyl ester	C_22_H_44_O_2_	57	340	0.82	–	–
37	33.91	**9,15-Octadecadienoic acid methyl ester**	C_18_H_32_O_2_	41	294	–	–	13.78
38	33.93	1-Hexadecanol	C_16_H_34_O	55	242	–	1.58	–
39	34.31	2-Nonadecanone	C_19_H_38_O	58	282	–	–	2.34
40	34.53	**Phytol**	C_20_H_40_O	71	296	38.28	52.10	19.39
41	34.96	Palmitaldehyde, diallyl acetal	C_22_H_42_O_2_	84	338	1.92	1.83	–
42	35.43	1-Propene-1,2,3-tricarboxylic acid, tributyl ester	C_18_H_30_O_6_	112	342	2.12	–	–
43	35.75	1-Hexacosanol	C_26_H_54_O	43	382	–	2.93	
44	35.76	10-Heneicosene	C_21_H_42_	55	294	2.51	–	–
45	35.85	Docosane	C_22_H_46_	43	310	0.63	–	0.58
46	36.28	Phytol acetate	C_22_H_42_O_2_	43	338	0.65	0.85	–
47	37.17	Tributyl acetylcitrate	C_20_H_34_O_8_	185	402	1.07	–	–
48	37.51	Tricosane	C_23_H_48_	57	324	1.33	–	–
49	37.58	Tridecane,5-methyl	C_14_H_30_	43	198	–	–	6.92
50	38.51	4,8,12,16-Tetramethyl heptadecan-4-olide	C_21_H_40_O_2_	99	324	–	1.11	0.5
51	39.03	1-Docosanol	C_22_H_46_O	43	326	–	2.27	
52	39.15	Hexatriacontane	C_36_H_74_	57	506	–	–	4.43
53	40.66	Pentacosane	C_25_H_52_	57	352	0.81	1.22	–
54	41.59	1,2-Benzenedicarboxylic acid, dioctyl ester	C_24_H_38_O_4_	149	390	0.71	–	–
55	42.41	Trans-Geranylgeraniol	C_20_H_34_O	69	290	–	–	0.39
56	43.62	Heptacosane	C_27_H_56_	57	380	–	–	1.10
57	45.49	Squalene	C_30_H_50_	69	410	1.17	–	–
58	48.92	Octacosane	C_28_H_58_	57	394	–	–	0.5
59	51.10	Stigmasta-5,22-dien-3-ol (Stigmasterol)	C_29_H_48_O	55	412	0.55	0.52	0.49
60	52.48	22,23-Dihydro stigmasterol (β-sitosterol)	C_29_H_50_O	43	414	0.45	0.41	0.5
61	52.88	Lupeol	C_30_H_50_O	43	426	0.82	0.13	1.82
Total identified compounds						99.27	99.33	97.50

*L* leaves, *S* stem, *F* flowers.

GC/MS analysis of saponifiable matter of *A. houstonianum* (Table 3, Fig. S3) revealed the identification of 16, 21 and 22 components representing 96.51%, 97.14% and 98.42%, of the total FAME of leaves, stems and flowers, respectively. It was observed that: The unsaturated fatty acids constitute the major makeup in the stem, leaves, and flowers (63.27, 56.25, 54.65%), respectively. Omegas 6 and 3 were detected in comparable amounts as the major makeup. On the other hand, palmitic acid was the major one (32.72%).

**Table 3:** GC/MS analysis of the fatty acid methyl ester matter (FAME) of *n*-hexane extract of the leaves, stems and flowers of *A. houstonianum*.

Peak no.	Rt (min)	Identified compound	Molecular formula	Base peak	Molecular ion peak (M^+^)	Yield (%)		
						L	S	F
1	5.97	9-octadecenoic acid, methyl ester **(Oleic acid, methyl ester)**	C_19_H_36_O_2_	55	296	–	13.66	–
2	7.97	Dodecanedioic acid, dimethyl ester	C_14_H_26_O_4_	55	258	–	5.65	–
3	8.58	Tetradecanoic acid, methyl ester (methyl myrictate)	C_15_H_30_O_2_	74	242	1.34	–	3.61
4	9.30	16-Octadecenoic acid, methyl ester	C_19_H_36_O_2_	55	296	–	–	0.14
5	10.25	Hexadecanoic acid, 15-methyl-, methyl ester	C_18_H_36_O_2_	74	284	–	0.28	–
6	10.75	Hexadecanoic acid,2,3-dihydroxypropyl ester	C_19_H_38_O_4_	55	330	0.14	–	–
7	10.80	Pentadecanoic acid, methyl ester	C_16_H_32_O_2_	74	256	–	–	0.28
8	10.86	Tetradecanoic acid, 12-methyl, methyl ester	C_16_H_32_O_2_	74	256	–	–	0.61
9	12.54	9-Hexadecenoic acid, methyl ester (methyl palmitoleate)	C_17_H_32_O_2_	55	268	0.49	–	0.47
10	12.65	13,16-Octadecadienoic acid, methyl ester	C_19_H_34_O_2_	55	294	–	–	0.95
11	13.10	Hexadecanoic acid, methyl ester **(palmitic acid, methyl ester)**	C_17_H_34_O_2_	74	270	32.72	22.74	34.27
12	13.24	5-Hexenoic acid, methyl ester	C_7_H_12_O_2_	74	128	–	–	9.39
13	14.77	Hexadecadienoic acid, methyl ester	C_17_H_30_O_2_	67	266	–	–	0.44
14	15.23	Hexadecenoic acid, 15-methyl, methyl ester	C_18_H_36_O_2_	74	284	0.38	–	0.83
15	15.69	Pentadecanoic acid, 14-methyl, methyl ester	C_17_H_34_O_2_	74	270	0.16	–	–
16	16.86	9,12-Octadecadienoic acid, methyl ester **(linoleic acid methyl ester)**	C_19_H_34_O_2_	67	294	29.13	24.40	15.29
17	16.93	9,12,15-Octadecatrienoic acid, methyl ester **(linolenic acid, methyl ester)**	C_19_H_32_O_2_	79	292	25.95	16.57	25.96
18	17.40	Octadecanoic acid, methyl ester (stearic acid, methyl ester)	C_19_H_38_O_2_	74	298	4.16	0.25	–
19	17.42	Heptadecanoic acid, 9-methyl, methyl ester	C_19_H_38_O_2_	74	298		0.82	–
20	18.26	9-Octadecenoic acid, ethyl ester	C_20_H_38_O_2_	55	310	–	0.98	–
21	18.31	1-Propene-1,2,3-tricarboxylic acid, tributyl ester (tributyl aconitate)	C_18_H_30_O_6_	112	342	0.49	–	–
22	18.81	Octadecanoic acid, ethyl Ester (Stearic acid, ethyl ester)	C_20_H_40_O_2_	88	312	–	2.46	–
23	18.95	4,7-Octadecadiynoic acid, methyl ester	C_19_H_30_O_2_	105	290	0.09	–	–
24	19.54	8-Methyl-9-tetradecen-1-ol acetate	C_17_H_32_O_2_	43	268	–	–	0.16
25	19.76	Oxiraneundecanoic acid, 3-pentyl, methyl ester	C_19_H_36_O_3_	55	312	–	–	0.27
26	19.90	6,9,12-Octadecatrienoic acid, methyl ester	C_19_H_32_O_2_	41	292	–	–	0.15
27	20.23	1,1′Bicyclopropyl-2-octanoic acid, 2′-hexyl, methyl ester	C_19_H_36_O_2_	73	322	–	–	0.77
28	20.44	Tributyl acetylcitrate (citroflex A)	C_20_H_34_O_8_	185	402	0.38	–	–
29	20.87	9-Octadecenoic acid, methyl ester (oleic acid, methyl ester)	C_19_H_36_O_2_	55	296	–	–	1.27
30	21.54	Eicosanoic acid, methyl ester (arachidic acid, methyl ester)	C_21_H_42_O_2_	74	326	0.57	–	2.05
31	21.56	Arachidonic acid, ethyl ester	C_22_H_36_O_2_	79	332	–	0.37	–
32	21.98	Hexadecanedioic acid, 3-methyl, dimethyl ester	C_19_H_36_O_4_	74	328	–	–	0.34
33	22.49	9,12,15-Octadecatrienoic acid, 2,3-dihydroxypropyl ester (1-mono linolenin)	C_21_H_36_O_4_	79	352	–	–	0.09
34	22.75	10-Heptadecen-8-ynoic acid, methyl ester	C_18_H_30_O_2_	79	278	–	5.17	–
35	25.42	Docosanoic acid, methyl ester	C_23_H_46_O_2_	74	354	0.29		0.84
36	25.87	1,2-Benzenedicarboxylic acid, dioctyl ester (dioctyl phthalate)	C_24_H_38_O_4_	149	390	0.1	0.35	–
37	26.52	8,11-Octadecadienoic acid, methyl ester	C_19_H_34_O_2_	67	294	–	1.05	–
38	29.23	17-Octadecynoic acid, methyl ester	C_19_H_34_O_2_	74	294	–	0.35	–
39	31.35	Triacontanedioic acid, dimethyl ester	C_32_H_62_O_4_	98	510	–	0.37	–
40	32.81	Docosanoic acid, methyl ester	C_23_H_46_O_2_	74	354	–	0.28	–
41	32.99	Tricosanoic acid, methyl ester	C_24_H_48_O_2_	74	368	–	0.34	–
42	33.31	15-Tetracosenoic acid, methyl ester	C_25_H_48_O_2_	55	380	–	0.37	–
43	33.59	Heneicosanoic acid, methyl ester	C_22_H_44_O_2_	74	340	–	0.34	–
Total identified compounds						96.51	97.14	98.42
Saturated fatty acids						40.26	33.87	43.77
Unsaturated fatty acids						56.25	63.27	54.65

### Larvicidal bioassay

The larvicidal activity of aerial parts of *A. houstonianium* extracts were evaluated against the 3rd instar larvae of *C. pipiens* at 24-, 48- and 72-post-treatment and the data represented in Table 4. The mortality rate of larvae increased with increase time of exposure and concentrations for all extracts. The results indicated that the extracts of *A. houstonianium* flower, leaf and stem influenced the mortality of larvae with LC_50_ values 259.79, 266.85 and 306.86 ppm, respectively, after 24 h of application. The flower extract showed high potency compared with leaf and stem extracts at the 1st, 2nd and 3rd day of exposure. The toxicity indexes of leaf and stem extracts decrease gradually with time. At 72 h post-treatment, the toxicity indexes of leaf and stem extracts were 73.91 and 59.10, respectively. The potency of flower and leaf extracts were 1.69 and 1.25 folds than stem extract, respectively. The slope values were low which indicate the homogeneity of the tested population.

**Table 4:** Larvicidal activity of *A. houstonianum* flower, leaf, and stem ethanol extracts on 3rd larval instar of *C. pipiens* 24, 48 and 72 h post-treatment.

Extract (ppm)	Flower			Leaf			Stem		
	24 h	48 h	72 h	24 h	48 h	72 h	24 h	48 h	72 h
LC_25_ (*F.l. at 95%)	159.40 (136.90–179.15)	117.34 (95.69–136.41)	89.22 (66.99–108.92)	133.72 (104.95–158.28)	122.65 (94.62–146.67)	113.01 (85.86–136.40)	158.083 (128.53–183.32)	166.74 (139.42–190.34)	144.24 (115.10–169.09)
LC_50_ (*F.l. at 95%)	259.79 (236.87–283.76)	203.07 (180.70–224.72)	168.043 (143.70–190.31)	266.85 (236.05–301.19)	246.33 (216.65–277.79)	227.34 (198.58–256.47)	306.86 (273.57–347.47)	302.42 (272.40–337.71)	284.31 (252.57–321.16)
LC_90_ (*F.l. at 95%)	657.16 (563.88–809.40)	575.69 (492.12–713.01)	559.488 (469.006–717.732)	991.71 (764.78–1472.53)	926.62 (720.53–1356.55)	857.91 (674.00–1233.78)	1082.13 (829.234–1622.945)	937.37 (748.39–1304.52)	1032.19 (793.75–1539.25)
Slope ± SE	3.17 ± 0.26	2.83 ± 0.26	2.45 ± 0.25	2.248 ± 0.255	2.22 ± 0.25	2.22 ± 0.25	2.34 ± 0.26	2.61 ± 0.27	2.28 ± 0.25
χ^2^	5.68	4.94	0.92	5.05	5.72	6.47	3.34	1.29	3.90
Probability (P)	0.13	0.18	0.82	0.17	0.13	0.09	0.34	0.73	0.27
Toxicity index	100	100	100	97.36	82.43	73.91	84.66	67.14	59.1
Relative potency	1.18	1.489	1.69	1.14	1.227	1.25	1	1	1

*(F.l.) Fiducially Limits *(χ^2^) Chi square value.

*Slope of the concentration-inhibition regression line ± standard error.

### Repellency/antifeedant action of *A. houstonianum* Mill. flower, leaf and seed extracts against the adult *Culex pipiens*

The overall, the repellency of the *A. houstonianum* flower, leaf and seed extracts tested and DEET gave a variable degree of repellency (Table 5). At a dose (1.8 mg/cm^2^), potent repellency (100%) was obtained by DEET through the 4 h post treatment, the other 3 extracts exhibited < 89.1% repellency within the 4 h post-treatment; the relative repellency was increased as the dose increased, where highest repellency % was obtained by flower extract (89.1%) at a dose 3.6 mg/cm^2^ decreased to 73.3% at a dose 1.8 mg/cm^2^ after 4 h from treatment, while the lowest repellency % was obtained by leaf extract (86.2%) at a dose 3.6 mg/cm^2^ decreased to 49.6% at a dose 1.8 mg/cm^2^ after 4 h post-treatment.

**Table 5:** Repellency/antifeedant effect of *A. houstonianum* Mill. (Asteraceae) flower, leaf and seed ethanol extracts on females of *C. pipiens*.

Plant parts	Dose (mg/cm^2^)	No. of tested females	No. of fed	%	No. of unfed	%	Repellency %
Leaf	3.6	48	6	12.5	42	87.5	86.3
	1.8	52	13	25.0	39	75.0	72.6
Flower	3.6	40	4	10.0	36	90.0	89.1
	1.8	42	11	26.2	31	73.8	73.3
Stem	3.6	33	10	30.3	23	69.7	68.2
	1.8	25	12	48.0	13	52.0	49.6
DEET	1.8	25	0.0	0.0	25	100.0	100.0
Control	–	23	21	91.3	2	8.7	0.0

### Biochemical activity

Activity of the enzymes, AChE, ATPase, CarE and CYP-450 were detected in the total hemolymph of the *C. pipiens* larvae treated with LC_50_ of *A. houstonianium* flower, leaf and stem extracts were shown in Table 6. AChE activity was significantly inhabited in *C. pipiens* larvae, the obtained inhibition ratios of enzymatic activity ranging from − 57.86% (flower), − 40.979% (leaf) to − 15.95% (stem). It was noticed that both flower and leaf extracts have high inhibition efficacy against acetylcholinesterase than stem extract.

**Table 6:** Effect of *A. houstonianium* flower, leaf, and stem extracts on the activity of acetylcholinesterase, carboxylesterase, ATPase, and cytochrome P-450 monooxegenase in 3rd larval instar of *C. pipiens.*

Enzyme	Activity mean ± SE			Control
	Flower	Leaf	Stem	
Acetylcholinesterase (ug AchBr/min/mg protein)	7.66 ± 2.7^a^ (− 57.86%)	10.73 ± 1.3^b^ (− 40.97%)	15.28 ± 1.05^c^ (− 15.95%)	18.18 ± 4.2^d^
Carboxylesterase (ug Meb/min/mg protein)	43.12 ± 10^a^ (− 29.32%)	47.303 ± 12.5^a^ (− 22.47%)	53.05 ± 5.5^b^ (− 13.04%)	61.01 ± 10.8^c^
ATPase (umoles Pi/min/mg protein)	78.81 ± 0.11^c^ (30.05%)	69.16 ± 0.10^b^ (14.13%)	63.93 ± 0.06^a^ (5.49%)	60.6 ± 0.12^a^
Cytochrome P-450 Monooxegenase (m mol sub. oxidized/min/mg protein)	37.16 ± 0.85^a^ (− 36.60%)	45.44 ± 2.05^b^(− 22.14%)	46.18 ± 0.98^b^(− 20.87%)	58.36 ± 2^c^

According to Duncan’s multiple range test (P ≥ 0.05), Means with the same letters are not significantly different. Each value represents the mean of three replicates ± SD, *SD* Standard deviation.

All tested extracts led to decrease in the amount of CarE which more obvious with flower extract than other extracts. It was 43.12, 47.30 and 53.05 (ug Meb/min/mg protein) for flower, leaf, and stem, respectively, as compared with control 61.01 (ug Meb/min/mg protein).

Results given in Table 6 indicated that the tested extracts increase the amount of ATPase which was clearly detected in flower extract treatment compared with control. Amount of ATPase were 78.81, 69.16 and 63.93 (umoles Pi/min/mg protein) for extracts of flower, leaf, and stem, respectively, while it was 60.6 (umoles Pi/min/mg protein) with control. A significant reduction in CYP-450 activity was obtained by treatment with all extracts whereas the flower extract showed the high reduction (− 36.606%) compared with leaf (− 22.14%) and stem (− 20.87%) extracts.

## Discussion

Chlorogenic acid is one of the most abundant beneficial polyphenols in plants and is well known as nutritional antioxidant in plant -based foods. Apart from its dietary antioxidant activity, it has been proven to be an efficient defense molecule against a broad range of insect herbivores^20^. Increased efficiency of bio-insecticides is achieved by using chlorogenic acid as a synergistic bacterium. Chlorogenic acid has chemical defense against insects ascribed to its prooxidant effect by binding of the highly reactive chlorogenoquinone with nucleophilic–NH_2_ and –SH groups in proteins and amino acids^21^. This reduces the bioavailability of amino acids consequently decreases digestibility of dietary proteins so, it considered as effective deterrent or anti-feedant^22^.

High performance liquid chromatography (HPLC) and quantitative determination of phenolic contents of *A. houstonianum* showed that the ethanolic flowers extract was the richest extract in the flavonoid and total polyphenolic contents followed by the leaves then the stems, which interpreted the high potency of flowers extract than leaves followed by stem. This high potency was due to the synergism of its bioactive compounds which detected in high levels than in leaves and stems extracts. Where, the flowers extract exhibited high activity against *C. pipiens* larvae with approximately 2-folds than leaves and stems. The same results were detected for repellency and antifeedant effects against the *C. pipiens* adults. Where the repellency % obtained by flower extract was (89.1%) at a dose 3.6 mg/cm^2^ indicating a good repellent property. Also, antifeedant activity and the maximum protection was obtained by flower extract with 90% of unfed females.

Regnault-Roger et al.^23^, showed that all phenolic compounds had toxicity to beetles, which paralyzed or dead at the bioassay test, by their cumulative toxic effect. Vanillin and caffeic and ferulic acids had a knockdown effect, while rosmarinic acid, gallic acid, naringin and luteolin-7-glucoside had significant toxic and attractive effects. Rosmarinic acid was also detected at high concentration in the flowers. Rosmarinic acid is an insecticidal agent with high insecticidal activity at very low concentrations in 24 h against aphids. Also, it is known to reduce genotoxic effects induced by harmful chemicals so, it considered very safe to consumers^24^. The flavonoid rutin negatively affected the behavior, biology, and physiology of *Spodoptera frugiperda* and *Helicoverpa zea* by prolonging the larval development time, reducing the larval and pupal weight, and decreasing the pupal viability. The addition of different concentrations of rutin prolonged the life cycle of *S. frugiperda*; therefore, the use of rutin is indicated in future studies evaluating the control of *S. frugiperda*^25^. The flower extract showed higher total identified flavonoids than leaves and stems. Flavonoids and iso-flavonoids adversely affect insect growth, development, and behavior by influencing the steroid hormone systems. Some flavonoids are highly toxic to insect, while other act as feeding deterrents and repellency property^25^. The coumarin exhibited acute toxicity and deteriorated the growth of red palm weevil larvae^26^ and showed antifeedant effects against *Rhyzopertha dominica* F. and *Oryzaephilus surinamensis* L. and demonstrated that the insect used the energy generated from ingested food to perform its physiological activities to fight the toxin (coumarin), therefore, affect the insect growth and development^27^. So, the polyphenols act in different ways and at different rate. Some components acted progressive toxicity while others had knockdown, repellent or anti-feedent effects.

Phytol was the major makeup in stem (52.10%), leaves (38.28%), and flowers 19.39%. Where, ketones represented by (4.65%, 4.69% and 5.12%) in the leaves, stems and flowers USM, respectively, the main of which was 2-pentadecanone, 6, 10, 14-trimethyl showing a yield of (2.05%) in the leaves and it is the only ketones present in the stems, while the main of which in flowers was 2-nonadecanone representing (2.34%). As well as aldehyde presented as (1.92% and 2.65%) in the USM of leaves and stems respectively, the main of which was palmitaldehyde diallyl acetal whose percentage was (1.83%) in stems and it is the only aldehyde detect in leaves. Furthermore, esters represented as (6.32%, 0.85% and 1.66%) in the leaves, stems and flowers USM, respectively. Acid and sterols were detected in comparable percent in the different organs under investigation. GC–MS analysis of the chloroform extract of *Ageratum conyzoides* whole plant prevailed 9,12-Octadecadienoic acid (12.48%), as major identified compound which comparable to our finding^28^.

Chromone presented by precocene II which was detected in leaves and stem as 22.08% and 13.26% respectively. While, in flower chromene I and II were detected. Chromone1 and 2 derivatives, detected in flower extract, are a well-known allelochemical and showed good insecticidal potency against *M. separata*^29^. Moreover, they have significant larvicidal activity against *C. pipiens*^30^. Also, these derivatives have antioxidant activity and MAOs inhibition activities^31^. These results agree with the present results, the flower extract exhibited higher insecticidal activity than stem and leaves against *C. pipiens* larvae.

Insecticidal effect of precocene II on the human body louse, *Pediculus humanus* was reported^32^. Essential oil of *A. houstonianum* Mill. aerial parts and its constituent compounds (precocene I and II) have potential for development into natural insecticides or repellents for control of insects in stored grains^33^. Precocene II inhibits juvenile hormone biosynthesis by cockroach corpora allata in vitro^34^. The precocenes (I and II), isolated from *A. houstonianum*, showed anti-juvenile hormonal effects on metamorphosis, ovarian development, and embryonic development also, exhibited larval mortality, the oviposition inhibition of ticks, Rhipicephalus microplus^35^. Fahmi et al.^36^, were investigate the influence of precocene II on the toxicological and biochemical parameters on the 4th instar larvae of *S. littoralis*. Overall, phytol can be considered further for developing effective and eco-friendly green insecticides against aphids^37^.

Whereas the ovicidal activity of *A. houstonianum* leaf extracts against the eggs of vector mosquitoes and to develop additional tools for the control of mosquito-borne diseases previously reported by Tennyson et al.^38^. The potential oviposition deterrent property of *A. houstonianum* crude leaf extracts detected in both laboratory and field studies designates the presence of phytocompounds that act as effective contact restraint^39^.

The insects have detoxification system to degrade toxic substances for the insect survival^40^. Metabolism of toxic substances involves two phases. The first phase is the cleavage of the substrate or addition of a polar group, while the second phase is the addition of sulfate, phosphate groups, sugar, or amino acid to the resulted products of 1st phase to increase hydrophilicity, consequently, facilitate excretion by the insect^41^. The most important enzymes responsible for the detoxification of toxins are CYP-450 for oxidative degradation and CarE for hydrolytic degradation that involved in 1st phase^42^. The detoxification capabilities of enzymes could be modified due to variations in gene expression^43^, consequently, variation of insect response to toxins^44^. The treatment of *C. pipiens* larvae with flowers, leaves and stem extracts inhibit the activity of CYP-450 and CarE activity with different levels due to variations in their constituents. The coumarin targets CYP-450 genes causing masking/silencing its expression that leads to high toxicity with low LD_50_ values against red palm weevil^26^. These results agree with^45^ who reported that the *Piper betle* extract reduced the level of CYP-450 in W strain of *Ae. aegypti*. Also, the sub-lethal dosage of *A. conyzoides* blocked the activity of CarE activity^46^. As well, the *Sophora alopecuroides* alkaloids are involved in the inhibition of CarE activity in *Aedes albopictus*^47^. In general, the esterases activities of the *H. armigera* larvae were significantly inhabited by flavonoid-treated diets^25^.

AChE has essential role as neurotransmitter in cholinergic synapses for insects^48^. Many insecticides inhibit of AChE action that causes accumulation of acetylcholine (ACh) at the synaptic cleft resulting in permanent neuro excitation/stimulation, paralysis, ataxia, and eventual death^49^. The obtained results showed that the flower and leaf extracts exhibited high inhibition effects against AChE than stem extract, that explained by Hussein et al.^30^, who proved that chromone 1 and 2 significantly inhibit the AChE activity in treated larvae of *C. pipiens* using molecular docking simulation. Many plant secondary metabolites decrease the levels of CarE and AChE activity of a wide range of insects^50^. The exposure of the *A. aegypti* larvae to the *Sapindus emarginatus* extract showed significant inhibition in the activities of AChE and CarE^51^, Similar reduction in AChE levels was observed by azadirachtin application against *Nilaparvata lugens*^52^.

ATPase plays a main role in intracellular functions and is a sensitive indicator of toxicity. It hydrolyzes adenosine triphosphate (ATP) to release the energy substantial for the active transport of Na^+^ and K^+^ across the cell membrane^53^. The metabolic detoxification mechanisms to toxins in insects consume high energy^54^. The elevated activity of the ATPase is a responsive action to the activation of detoxification mechanisms as a defense mechanism therefore, high energy demands^55^. Toxicity of botanical toxins to insects has been associated with the overexpression of genes involved with ATPase synthesis and energy demand^56^, this concept interpreted the enhancement of ATPase activity to reduce the damage caused by flower and leaves extracts, respectively, while the stem extract did not greatly stimulate ATPase with low expression.

Plant extracts have been studied extensively for their insecticidal effect^57^. Phytochemicals such as phenolic acid, flavonoids, chromene, phytol and monoterpenes are known for their mosquito repellent and insecticidal properties^57^. *Ageratum houstonianum* essential oil and extracts have been stated to have bioactive molecules^58^ with repellency and adulticidal action against the adult mosquitoes^59^. There are various degrees of activity of *Ageratum* sp. extracts against insects due to variation of active ingredients with a wide variety of insecticidal properties^60^ which agree with the results obtained in our investigation.

Many publications on the phytochemistry of *Ageratum sp.* from many disparate countries have been dealt with the various extracts with diversity in major and minor active constituents^61^. Petroleum ether extract of *A. conyzoides* showed significant larvicidal activity against the 4th larval instars, adult mortality and affected percentages of oviposition deterrence index of females of three mosquito vectors. Beside to, these extracts harmless to aquatic mosquito predator *Toxorhynchites splendens* even at the prominent dosage (1000 ppm)^46^. The *A. conyzoides* ethanolic extract has acaricidal potency against acaricides- susceptible and resistant ticks infesting buffaloes and cattle, moreover, adversely affected egg laying capacity^35^.

## Materials and methods

### Plant material

One kilogram of leaves, stems, and flowers of *A. houstonianum* Mill., collected individually during the flowering season from April to September 2019 from herbs growing in El Orman botanical garden, Giza square https://goo.gl/maps/NnGubZ5FDnE8RJZX8. The plant was authenticated by Dr. Reem Sameer, Professor of Plant Taxonomy, Botany Department, Faculty of Science, Cairo University. A voucher specimen (No. 26. 3.2018) was kept at the Herbarium of Pharmacognosy Department, Faculty of Pharmacy, Cairo Universityhttps://goo.gl/maps/v6PsvJp6KJW52PkH8. The use of plants in the present study complies with international, national and/or institutional guidelines.

### Preparation of plant extract

One hundred grams of the powdered leaves, stems, and flowers of the plant were separately extracted with about 1000 ml of 70% ethanolic solution by using maceration till exhaustion then filtered. The collected extract was completely dried under vacuum using rotatory evaporator at 40 °C to yield a residue of about 30 g, 15 g and 25 g extracts for leaves, stems, and flowers, respectively. The extract was kept in tightly sealed containers to be used for the polyphenolic and biological study.

### Preparation of the n-hexane extracts

The powdered dried leaves, stems, and flowers (1000 g, 165 g and 150 g, respectively) of *A. houstonianum* were exhaustively extracted in a Soxhlet apparatus with *n*-hexane. The extracts were evaporated under reduced pressure at 40 °C to yield (35 g, 2 g and 7 g) greasy, dark green residue of leaves, stems, and flowers, respectively. The residues were stored in a desiccator for lipoidal matter investigation.

### Preparation of the lipoidal matters

The lipoidal matters; unsaponifiable matter (USM) and fatty acid methyl esters (FAME) were prepared according to the method of Ichihara and Fukubayashi^62^, to identify the lipoidal constituents and to determine their percentages in the *n*-hexane extracts of leaves, stems, and flowers of *A. houstonianum*.

### Spectrophotometric determination of total phenolic contents

The polyphenol content was determined using the Folin-Ciocalteu reagent method according to Mruthunjaya and Hukkeri^63^, with some modifications. The method involves the reduction of Folin Ciocalteau reagent (Sigma chemical, St.louis, Missouri, USA) by phenolic compounds, with a concomitant formation of a blue complex, and the absorbance was read at 765 nm using an UV–Vis spectrophotometer. The total polyphenolic content was expressed as gallic acid, using a standard calibration curve. Each experiment was repeated in triplicate and the readings were mean values. Same practice was repeated for the standard solution of gallic acid, and the calibration line was constructed. Based on the absorbance, the concentration of phenolics was interpreted (mg/ml) from the calibration line; then the contents of phenolics in extracts were articulated in the total phenolic contents as gallic acid correspondent (mg of GAE/g of sample).

### Spectrophotometric determination of total flavonoid contents

Total flavonoid content was determined according to Atanassova et al*.*^64^, with some modifications. The absorbances of the solutions were measured at 510 nm against blank using a spectrophotometer. Similar procedure was returned for the standard solution of quercetin and the calibration graph was constructed. The content of flavonoids in each sample was articulate as quercetin, using a standard calibration curve as mg of QAE/g of sample).

### HPLC analysis of the phenolic components

HPLC quantitative analysis of phenolic components was performed according to method presented by Mizzi et al*.*^65^. Using an Agilent 1100 series LC System) equipped with a model G 1311 A quaternary solvent pump and degasser, a thermostatted column compartment (G1316A), autosampler (G1329A) and a diode array detector—DAD (G1315B). The analytical column was Eclipse XDB-C18 (150 × 4.6 μm; 5 μm) with a C18 guard column (Phenomenex, Torrance, C.A.). Mobile phase: The mobile phase consisted of acetonitrile (solvent A) and 2% acetic acid in water (v/v) (solvent B). Gradient programmed as follows: 100% B to 85:15 B: A, v/v in 30 min. 85:15 B: A to 50:50 B: A in 20 min, 50:50 B: A in 5 min, 0:100 B: A in 5 min and 100% A to 100% B in 5 min. Injection volume:50 μl, Flow rate:0.8 ml/min. Column temperature 30 °C. Detector type DAD detector, wave length 280 and 330. For investigations of phenolic acids and flavonoids, National Research Center. Phenolic acid and standards from Sigma Co. were dissolved in the mobile phase and injected into HPLC. Peaks were integrated both manually and using Agilent software. Retention time and peak area were used to calculate phenolic acids and flavonoids concentrations by data analysis using Agilent software The data collect and analyses were carried out using the software ChemStation Rev. A.10.02 Edition (copyright Agilent Technologies, 1990–2003.

### GC–MS analysis of lipid constituents

The prepared USM and FAME were analyzed by GC–MS. Using Thermo Scientific, trace GC Ultra/ISQ Single Quadrupole: MS, TG-5MS fused silica capillary column, coupled to an electron ionization system, for analysis of lipoidal content, National Research Center. The GC/MS analysis of the unsaponifiable and saponifiable fractions obtained from the powdered dried leaves, stems and flowers was carried out adopting the following conditions column typeTG-5MS fused silica capillary column. Column internal diameter 30 m, 0.251 mm, 0.1 mm film thickness. Carrier gas is Helium. Flow rate 1 ml/min. Sample size 1 μl. Injection mode: split less. Temperature programming in USP 50 °C (2 min) then elevated to 150 °C at a rate of 7 °C/min then to 270 °C at a rate of 5 °C/min (hold for 2 min) then to 310 °C at a rate of 3.5 °C/min and isothermally 10 min. In FAME temperature programming is 50 °C (4 min) then elevated to 280 °C at a rate of 5 °C/min and isothermally for 4 min. Injector temperature 280 °C. Ionization voltage70 eV. Scan mass range 50–500 m/z. Identification of the components was achieved by library research database, Wiley mass spectral database and by comparing their retention indices and mass fragmentation patterns with those of the available references as well as, published data^28^.

### Insect rearing

#### Maintenance of mosquito colony

The laboratory strain of *C. pipiens* was reared and maintained continuously for several generations in an insectary in Research and Training Center for Vectors of Diseases (RTC), in Faculty of Science, Ain Shams University, using the standard procedures described by Kasap and Demirhan^66^, under controlled conditions; 27 ± 2 °C and RH 75 ± 5%, and photoperiod 12:12 light: dark hours^7^. The newly hatched larvae were fed on Tetramin. The pupae were collected and transferred to the rearing screened wooden cages (25 × 25 × 25 cm). Adults were provided daily with a 10% sucrose solution. The females were allowed to feed a blood meal from a pigeon host.

#### Larvicidal bioassay

The 3rd arval instar of *C. pipiens* was treated with serial concentrations of *A. houstonianum* flower, leaf and stem extracts according to the previous standard protocol^67^ with some modifications. Five concentrations of *A. houstonianum* flower, leaf and stem extracts were prepared in ethanol for stock solution, while serial concentrations (500, 400, 300, 200 and 100 ppm) were diluted using distilled water to prepare 100 ml of each concentration. Distilled water only was used for control. Twenty larvae were transferred to each treatment and control. Each treatment and control were replicated three times. Mortality was recorded after 24-, 48- and 72-h post-treatment.

#### Repellency and antifeedant bioassay

The standard cages (20 × 20 × 20 cm) were used to test the repellent activity of the extracts. Different amounts from each extract were dissolved in 2 ml (distilled water with a drop of Triton × 100) in 4 × 4 cm cups to obtain the different concentrations. The concentration was directly applied onto 5 × 6 cm of the ventral surface of pigeon after removing the abdomen’s feathers. After 10 min of treatment, pigeons were placed for 3 h (from 6 to 9 PM) in cages containing the laboratory strain of starved *C. pipiens* females. Control tests were carried out using water. Each test was repeated three times to get a mean value of repellent activity^68^. Post treatment, the number of fed and unfed females was counted, and repellency was recorded statistically by using Abbott formula^69^.

$$ {\text{The repellency }}\% = \, \left( {\% {\text{ A}} - \% {\text{ B}}/{1}00 - {\text{B }}\% } \right) \times {1}00, $$

where A: the percentage of unfed females in treatment. B: the percentage of unfed females in control.

### Biochemical analysis

#### Enzyme preparation

The whole 3rd instar larvae of *C. pipiens* treated with LC_50_ values were homogenized in distilled water (50 mg/1 ml). Homogenates were centrifuged at 8000 r.p.m. for 15 min at 5 °C in a refrigerated centrifuge. The deposits were discarded, and the supernatants were kept in a deep freezer (2 °C) till use as Amin^70^.

#### Acetylcholinesterase (AChE) activity assay

Acetylcholine bromide (AChBr) was used as substrate to detect the AChE activity according to the method described by Simpson et al*.*^71^. 200 µl enzyme solution were mixed with 0.5 ml AChBr (3 mm) and 0.5 ml 0.067 M phosphate buffer (pH 7). The mixture tubes were incubated for 30 min at 37 °C. Then 1 ml of alkaline hydroxylamine and 0.5 ml of HCl were added. The mixture tubes were mixed well and allowed to stand for 3 min. 0.5 ml of FeCl_3_ solution was added to the mixture tube and shaken vigorously. The decrease in AChBr level resulted from the hydrolysis by AChE was read at 515 nm.

#### ATPase activity assay

The total ATPase activity was estimated as described by Amaral et al*.*^72^. The main concept of this method is estimation the amount inorganic phosphate (Pi) resulted from ATP hydrolysis by ATPase. The enzyme was incubated at pH 7.5 and 37 °C, in 0.5 ml of a solution containing mixture of NaCl 150 mM, ATP.Na2-TRIS 5 mM and KCl 15 mM in histidine HCl-TRIS 30 mM. ATP was added to start the reaction. The mixture was incubated for 30 min at 37 °C, then 100 μl SDS (5%) was added to stop the reaction. The amount of formed Pi was measured by phosphorus kit. ATPase activity was expressed in µmoles of Pi released per minute per milligram protein.

#### Cytochrome P-450 monooxegenase (CYP-450) activity assay

*P*-nitroanisole *O*-demthylation was used to determine the CYP-450 activity according to Hansen and Hodgson^73^ method with some modifications. The mixture solution containing 1.5 ml enzyme solution, 0.2 ml NADPH, 1 ml sodium phosphate buffer (0.1 M, pH 7.6), 50 µg glucose-6-phosphate dehydrogenase and 0.2 ml glucose-6-phosphate. p-nitroanisole in 10 µl of acetone was added to start the reaction and attain the final concentration of 0.8 mM. The final mixture was incubated at 37 °C for 30 min then 1 ml HCl (1 N) was added to terminate the incubation period. p-nitrophenol was extracted with 0.5 N NaOH and CHCl_3_. The absorbance of NaOH solution was estimated at 405 nm. An extinction coefficient of 14.28 mM/cm was used to calculate 4-nitrophenol concentration.

#### Carboxylesterase (CarE) activity assay

Carboxylesterase activity was determined as described by method of Simpson et al*.*^71^, and methyl n butyrate (MeB) used as substrate. The reaction solution containing 0.5 ml MeB (4 mM), 200 µl enzyme solution and 0.5 ml 0.067 M phosphate buffer (pH 7). The mixture tubes were incubated for 30 min at 37 °C. Then, 1 ml of alkaline hydroxylamine (equal volume of 3.5 M NaOH and 2 M hydroxylamine chloride) was added to the mixture tubes followed by 0.5 ml of HCl. The mixture tubes were mixed well and allowed to stand for 3 min. 0.5 ml of FeCl_3_ solution was added to the mixture tube and shaken vigorously. The decrease in MeBr level resulted from the hydrolysis by carboxylesterases was read at 515 nm.

### Statistical analysis

Lethal concentrations were determined at the 95% confidence level were recorded in probity regression line and LC_50_, and LC_90_, slope, standard error, and correlation coefficient; and for the goodness of fit (*Chi square test*) were calculated according to Finney^74^ and correction for control mortality was conducted using Abbott’s formula according to Abbott^69^. The biochemical results were analyzed by one-way analysis of variance (ANOVA) using CoStat system for Windows, Version 6.311 (CoHort software, Berkeley, CA 94701) https://www.cohortsoftware.com/costat.html. When the Anova statistics were significant (P < 0.01), means were compared by the Duncan’s multiple range test^75^.

## Conclusion

Overall, results suggest that the ethanolic ratios of enzymatic activity ranging extracts of flower, leaves, and stem of *A. houstonianum* exhibited a significant repellent, antifeedant and larvicidal activities with different levels, which may be attributed to chlorogenic, phytol, coumarin, rosmarinic acid, rutin, precocene I, and II compounds. All these bioactive molecules act in different ways with various rates and synergist each other to exhibit the toxicity action. Some components acted progressive toxicity while others had knockdown, repellent or anti-feedent effects. The flowers extract was rich with bioactive components which responsible for its high efficacy relative to leaves and stem extracts. The tested extracts inhibited the activity of AChE, CYP-450 and CarE with various levels, while the ATPase activity was enhanced. Different organs of *A. houstonianum* ethanol extracts could be used as bio-agents for mosquito control.

## Supplementary Information

Supplementary Figure S1.

Supplementary Figure S2.

Supplementary Figure S3.

## Data Availability

The datasets used and/or analysed during the current study available from the corresponding author on reasonable request.
