# Supplementary figures and images for: Larvicidal and repellent potential of Ageratum houstonianum against Culex pipiens
Source: Sci Rep. 2022 Dec 10;12:21410. doi: 10.1038/s41598-022-25939-z (PMC9741651; doi:10.1038/s41598-022-25939-z)

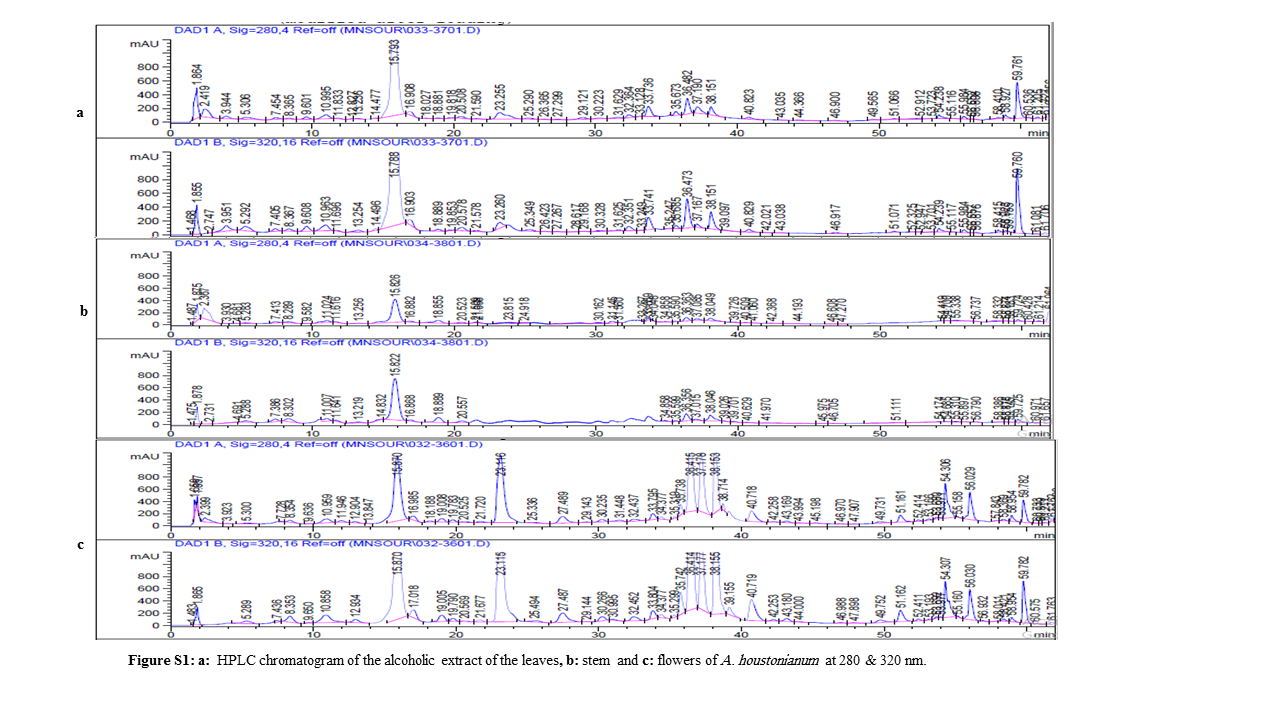

Supplement: Supplementary file 1 — Supplementary Figure S1. [file 41598_2022_25939_MOESM1_ESM.tif]

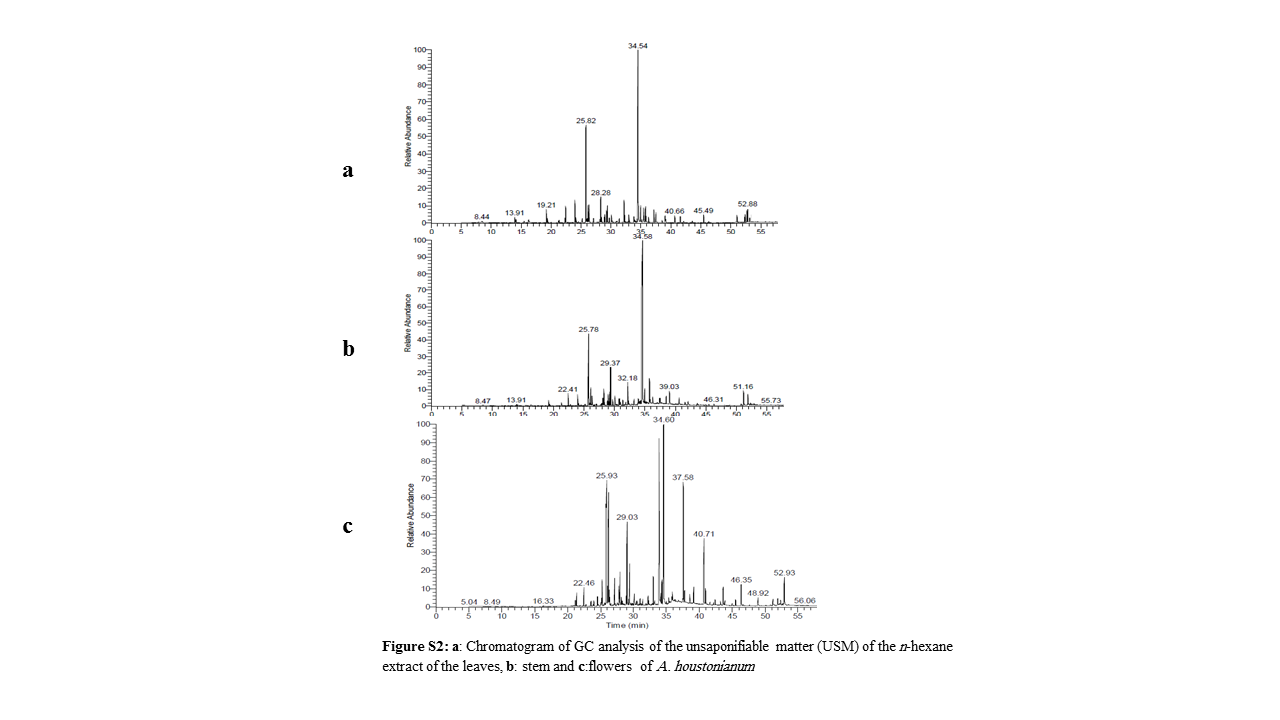

Supplement: Supplementary file 2 — Supplementary Figure S2. [file 41598_2022_25939_MOESM2_ESM.tif]

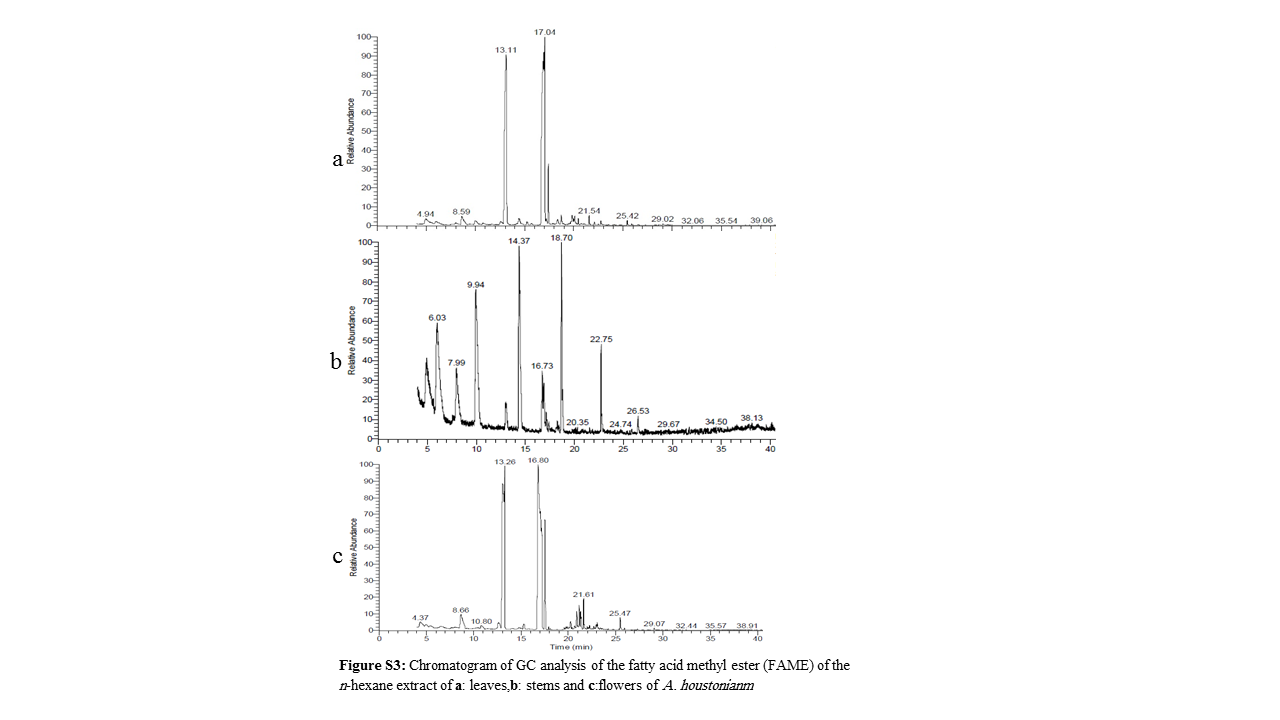

Supplement: Supplementary file 3 — Supplementary Figure S3. [file 41598_2022_25939_MOESM3_ESM.tif]
